# Fecal Elastase-1 in Healthy Children Up To 2 Years of Age: a Cross-sectional Study

**DOI:** 10.34763/devperiodmed.20182202.123127

**Published:** 2018-06-30

**Authors:** Mirosława Wieczorek-Filipiak, Sławomira Drzymała-Czyż, Mariusz Szczepanik, Anna Miśkiewicz-Chotnicka, Ewa Wenska-Chyży, Jerzy A. Moczko, Jarosław Walkowiak

**Affiliations:** 1 Department of Pediatric Gastroenterology and Metabolic Diseases, Poznan University of Medical Sciences, Poznań, Poland; 2 Department of Computer Science and Statistics, Poznań University of Medical Sciences, Poznań, Poland

**Keywords:** fecal test, exocrine pancreatic function, pancreatic function test, healthy infants, badanie kału, funkcja zewnątrzwydzielnicza trzustki, test funkcji trzustki, zdrowe niemowlęta

## Abstract

**Background:**

Fecal elastase-1 (E-1) levels in infants and young children may be expected to differ from those in adults and older children because of the immaturity of the gastrointestinal tract and the specificity of their diet. Despite the availability of data describing E-1 levels in the stools of preterm infants, older children, adults and subjects with malabsorption, there is still a lack of data regarding E-1 in healthy infants and toddlers.

The aim of this cross-sectional study was to evaluate fecal E-1 concentrations in infants and children from 1 up to 24 months of age.

**Material and methods:**

E-1 was measured in 160 healthy subjects aged 1-24 months (8 groups of 20: aged 1-3, 4-6 months, etc.) using an enzyme-linked immunosorbent assay (ELISA).

**Results:**

Fecal E-1 concentrations ranged from 200 to 1695 μg/g of feces. No child had a fecal E-1 level below 200 μg/g of feces. Fecal E-1 concentrations did not significantly differ between age groups. However, fecal E-1 levels in the first 3 months were lower than in the second year of life (1-3 months vs 13-24 months, p=0.0230). A statistically significant correlation between the E-1 concentration and age was found (p=0.0007, r=0.2639; however, it does not affect the cut-off level of the reference values). The trend was rather exponential. Fecal E-1 values reached a plateau around the age of 6-10 months.

**Conclusions:**

Our study has shown that the fecal E-1 test can be reliably applied in infants and toddlers to confirm normal exocrine pancreatic function. However, within the first months of life fecal E-1 concentrations may be lower than later in life.

## Streszczenie

**Wstęp:**

Stężenie elastazy-1 (E-1) w kale u niemowląt i małych dzieci może znacząco różnić się od wartości obserwowanych u osób dorosłych i dzieci starszych, co związane jest z niedojrzałością przewodu pokarmowego i swoistą dietą. Pomimo dostępnych danych opisujących poziomy E-1 u niemowląt urodzonych przedwcześnie oraz starszych dzieci, osób dorosłych i pacjentów niedożywionych, wciąż brakuje badań dotyczących poziomu E-1 u zdrowych noworodków i niemowląt.

Celem badania było określenie stężenia E-1 w stolcu w tej grupie wiekowej od 1-24 miesiąca życia.

**Materiał i metody:**

U 160 zdrowych dzieci w wieku 1-24 miesięcy (8 grup po 20 dzieci: w wieku 1-3, 4-6, 7-9, 10-12, 13-15, 16-18, 19-21, 22-24 miesiące) zmierzono stężenie E-1 w kale z zastosowaniem testu immunoenzymatycznego (ELISA).

**Wyniki:**

Stężenia E-1 wynosiły od 200 do 1695 μg/g kału. U żadnego dziecka nie odnotowano wartości E-1 poniżej 200 μg/g kału. Stężenia E-1 nie różniły się znacząco pomiędzy poszczególnymi grupami wiekowymi. Jednak w pierwszych 3 miesiącach poziomy E-1 były mniejsze niż w drugim roku życia (1-3 vs. 13-24 miesiące, p=0,0230). Stwierdzono istotną statystycznie korelację pomiędzy stężeniem E-1 a wiekiem dziecka (p=0,0007, r=0,2639; nie ma ona jednak wpływu na punkt odcięcia wartości referencyjnych). Tendencja ta miała raczej charakter wykładniczy. Wartości E-1 w kale osiągnęły plateau w wieku 6-10 miesięcy.

**Wnioski:**

Potwierdzenie prawidłowej funkcji zewnątrzwydzielniczej trzustki może być dokonywane u niemowląt i małych dzieci poprzez pomiar stężenia E-1 w kale. Jednakże w ciągu pierwszych miesięcy życia poziomy E-1 w stolcu mogą być niższe niż w późniejszym okresie życia.

## Introduction

Elastase-1 (E-1) is a marker of exocrine pancreatic insufficiency that is widely used owing to its high specificity and sensitivity [1, 2, 3, 4, 5]. Low fecal E-1 concentrations occur in diseases involving pancreatic dysfunction, such as: cystic fibrosis, diabetes, chronic pancreatitis, pancreatic cancer, coeliac disease and human immunodeficiency virus infection [6, 7, 8, 9, 10].

The abundance of E-1 in stools depends on a person’s diet and age. Our previous research revealed that abstaining from meat results in a significant decrease in E-1 [11, 12] and that E-1 levels in the elderly are lower compared with young people, reflecting the natural aging of the pancreas [13]. Many different changes may be expected to be found in infants and young children since their gastrointestinal tract is immature and their diet differs from that of adults. However, the available data regarding fecal E-1 concentrations in healthy children, and especially in infants, is scarce. In fact, most of the studies conducted to-date described the evolution observed in the secretion of pancreatic enzymes over the first days after birth in preterm infants and measuring E-1 levels in their meconium [14, 15, 16]. Low E-1 concentrations in the meconium and their increase in feces in the first weeks of life was noted in these studies. However, research which would involve representative groups of healthy infants and toddlers is still lacking.

## Aim

The aim of this cross-sectional study was to evaluate fecal E-1 concentrations in infants and children from 1 up to 24 months of age.

## Materials and methods

### Patients

The study comprised 160 healthy subjects (82 boys, 78 girls; aged 1 to 24 months old). They were assigned into eight age groups: 1-3, 4-6, 7-9, 10-12, 13-15, 16-18, 19-21, 22-24 months (n=20 in every group).

The inclusion criteria were: age of 1 to 24 months, good general status, normal way of feeding, willingness to participate in the study. The exclusion criteria were: prematurity, failure to thrive, diagnosed disease (e.g., chronic gastrointestinal diseases, inflammatory processes).

All the children remained under the care of the investigator (MFW) for at least three years after E-1 was measured. No signs and symptoms of neither exocrine pancreatic insufficiency or other gastrointestinal diseases potentially influencing pancreatic secretion have been observed in any of the study participants.

The protocol of the investigation was approved by the Ethical Committee of Poznań University of Medical Sciences, Poznań, Poland (decision no. 1275/05). Written informed consent was obtained from all the children’s parents. The study was carried out in accordance with the revised Declaration of Helsinki.

### Methods

In every child the *Z-score* for body weight was calculated [17]. Fecal E-1 concentrations were measured in stool samples with a commercially available monoclonal antibody kit (ELISA; ScheBo BioTech, Giessen, Germany) [6,18]. Results were expressed as μg/g of feces.

### Statistical methods

All the statistical analyses were performed in the Statistica 12.0 software environment (StatSoft, Inc., Tulsa, USA) and Stata/IC 15.0 64 bit for Windows (StataCorp LP, Lakeway Drive, USA). Normality was determined using the Shapiro-Wilk test. The normal distribution mean and standard deviation (SD) are reported for variables. Medians and 1st–3rd quartiles are given for non-normal variables. Differences between multiple groups were assessed using the Kruskal-Wallis test and one-way analysis of variance with post-hoc testing (Bonferroni-corrected). The linear correlation between E-1 concentrations and age were analyzed using Pearson’s test.

E-1 results were smoothed for graphic presentation using the LOWESS method (Stata/SE 15.0 64 bit for Windows, Tulsa, USA).

Values of p<0.05 were considered to be statistically significant.

## Results

The anthropometric parameters describing the subjects are presented in Table 1. No statistically significant differences between *Z-scores* for body weight and sex were found between the groups (Table I).

Fecal E-1 concentrations ranged from 200 to 1695 μg/g of feces. No child had fecal E-1 levels below the cut-off level (200 μg/g) suggestive of abnormal exocrine pancreatic function.

**Fig. 1:**
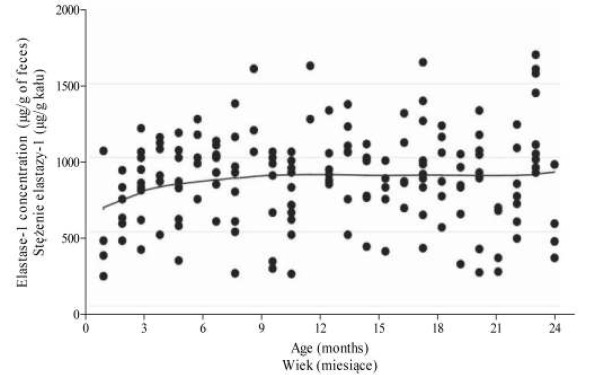
Scattergram presenting the dependence of elastase-1 concentration on age − results smoothed using the LOWESS method (bandwidth = 0.8). Ryc. 1. Zależność stężenia elastazy-1 względem wieku wygładzona przy użyciu metody LOWESS (szerokość pasma = 0.8).

Fecal E-1 concentrations did not significantly differ between age groups (Table II). However, fecal E-1 levels in the first 3 months were lower than in the second year of life (median [1^st^-3^rd^ quartile], 790 μg/g [527-920] vs 900 μg/g [728-1070]; p=0.0230). A statistically significant correlation between E-1 concentration and age was found (p=0.0007, r=0.2639; however, it does not affect the cutoff level of the reference values). The trend observed was rather exponential (Figure 1). Fecal E-1 values reached a plateau around the age of 6-10 months.

## Discussion

The topic of our study was the evaluation of fecal E-1 concentrations in healthy children aged 1-24 months. This has been the first time when in such a large cohort of healthy infants and young children (n=160) exocrine pancreatic function was studied using reliable indirect pancreatic function tests. It should be emphasized that this is the first study to create reference values of E-1 in healthy infants and toddlers.

E-1 concentrations in all children were within the normal range for adults. Similarly, Nissler *et al*. reported that 96.8% of the infants they studied had higher fecal E-1 levels after the second week of life than the adult lower limit of normal values, independent of the gestational age at which they were born [16]. However, their study comprised predominantly patients younger than 3 months with only a few older infants. It should be noted that the youngest child in the present study was older than 4 weeks and no meconium assessments were made. We did not include neonates, because extensive data are already available for this age group [14, 15, 16,19, 20, 21]. Kori *et al*. noted that in all the samples analyzed (n=63) regardless of gestational age, the E-1 level was below normal range (200 μg/g of feces; mean (SD) 45.9 μg/g of feces (51.1)) and found that E-1 normalization followed day 3 in term newborns [14]. Also in preterm babies studies described a low E-1 concentration in their first few days of life (median (range): 89 μg/g of feces (3-539) at day 2) which gradually increased on subsequent days (median (range): 164 μg/g of feces (3–600 at day 5) [15].

In our study fecal E-1 levels were lower within the first months of life. Later they reached a plateau corresponding to normal adult levels. Also Terbrack *et al*. described a slight tendency of E-1 levels to increase with age and similarly high enzyme concentrations in a population of older infants and children (the mean was 763 μg/g) [19]. However, a detailed analysis was performed only for the first 4 months of life (4 groups aged 1, 2, 3 and 4 months). Older subjects were divided into 2 groups with a large age span (the first group − from 5 months to 1 year, the second − from 1 year to 14 years).

The increase of values observed within the first months of life in the present study may be due to the development of the pancreas. Although the pancreas begins to produce enzymes already in the 20^th^ week of gestation, newborns secrete fewer pancreatic enzymes than older children (for example, amylase levels are undetectable, while lipase is produced on a level of less than 10% of childhood levels) [22, 23]. It should be noted that the ontogeny of E-1 in infants is still unknown.

**Table I:** Basic anthropometric data of healthy infants and young children. Median values [1^st^-3^rd^ quartiles] are presented. Tabela I. Podstawowe dane antropometryczne zdrowych niemowląt i małych dzieci. Przedstawiono wartości mediany oraz [I-III kwartyl].

	Age (months) *Wiek (miesiące)*								
	1-3	4-6	7-9	10-12	13-15	16-18	19-21	22-24	*p*
*Z-score* for body weight *Z-score dla masy ciała*	-0.03 [-0.46 -1.04]	-0.05 [-0.90 -0.85]	0.14 [-0.49 -0.55]	0.09 [-0.44 -0.65]	-0.14 [-0.73 -0.47]	0.08 [-0.35 -0.89]	0.02 [-0.92 -0.53]	0.01 [-0.61 -0.43]	Ns.
Sex ratio (male/female) *Stosunek płci* *(mężczyźni/kobiety)*	8/12	10/10	10/10	10/10	7/13	11/9	14/6	12/8	Ns.

The number of children in each group was 20 W każdej grupie wiekowej było 20 dzieci

**Table II:** Elastase-1 concentration (μg/g of feces) in healthy infants and young children. Mean values and (± standard deviation) are presented. Tabela II. Stężenie elastazy-1 (μg/g kału) u zdrowych niemowląt i małych dzieci. Przedstawiono średnie wartości oraz (± odchylenie standardowe).

	Age (months) *Wiek (miesiące)*								*p*
	1-3	4-6	7-9	10-12	13-15	16-18	19-21	22-24	
Elastase-1 concentration (μg/g of feces) *Stężęnie elastazy-1* *(μg/g kału)*	724.9 (±272.6)	883.8 (±256.3)	932.4 (±309.9)	793.0 (±344.7)	934.3 (±244.0)	911.3 (±313.6)	844.4 (±305.4)	933.0 (±417.5)	Ns.

The number of subjects in each group was 20 W każdej grupie wiekowej było 20 dzieci

The documented differences in fecal E-1 concentration between age subgroups might be related to weaning and expanding infants’ diet (especially by the 6^th^ month of life). In the data available, the normal levels of fecal E-1 were found sooner in infants who started enteral feeding earlier [14, 15]. It should, however, be noted that infants are fed a very specific (high fat) diet based exclusively on milk fat. On the other hand extending their diet may stimulate enzyme production and secretion, which is supported by studies on adults that have shown that an abstinence from meat may lead to a decrease in fecal E-1 concentration [11].

It should be stressed that the abundant data concerning fecal E-1 concentrations in children with cystic fibrosis [24, 25, 26, 27] or preterm infants [14, 15, 21] cannot be extrapolated from healthy infants. All the studies conducted on healthy children so far involved heterogenous age groups (from birth to almost adulthood) with low numbers of infants and toddlers [28, 29]; this renders the results virtually uninterpretable beyond the neonatal and early post-neonatal period.

Although the present study comprises a large and representative group of infants and young children aged up to 24 months, it also has some limitations which includes the cross-sectional character of the study with the lack of longitudinal follow-up of fecal E-1 concentrations, the absence of information on the weaning status, and the lack of correlation of fecal E-1 levels to energy and nutrient intake.

## Conclusions

Our study has shown that the fecal E-1 test can be reliably applied in infants and toddlers to confirm normal exocrine pancreatic function. However, within the first months of life fecal E-1 concentrations may be lower than later in life.
